# Risk determinants associated with early childhood caries in Uygur children: a preschool-based cross-sectional study

**DOI:** 10.1186/1472-6831-14-136

**Published:** 2014-11-18

**Authors:** Jibieke Wulaerhan, Ayinuer Abudureyimu, Xue-Li Bao, Jin Zhao

**Affiliations:** Department of Endodontics, The First Affiliated Hospital of Xinjiang Medical University, Urumqi, Xinjiang 830054 People’s Republic of China; Stomatology Disease Institute of Xinjiang Uyghur Autonomous Region, Urumqi, Xinjiang 830054 People’s Republic of China

**Keywords:** Early childhood caries, Risk indicators, Uygur ethnic minority

## Abstract

**Background:**

The prevalence of early childhood caries (ECC) varies with geographical region and population. The Uygur people, one of 55 officially recognized ethnic minorities in China, have a population of 10,069,346. We performed a preschool-based cross-sectional study of 670 Uygur children from the southern region of Xinjiang, China, to investigate the prevalence and severity of ECC and to identify factors related to the dental health condition of this population.

**Methods:**

The study population of children ranging in age from 3 to 5 years was invited using a three-stage stratified sampling in Kashgar, the westernmost city in China. The “dmft” index was used to assess dental caries. The diagnosis of ECC or severe ECC was based on the oral health diagnostic criteria defined by the American Academy of Pediatric Dentistry. A questionnaire was completed by the children’s caregivers. The survey included questions concerning the children’s sociodemographic background; feeding and eating habits, particularly frequency of sweet beverage and food consumption; dental hygiene-related behaviors; the general oral health knowledge of caregivers; and the dental healthcare experience of caregivers and their children.

**Results:**

A total of 670 Uygur children underwent complete dental caries examination. Most of the children (74.2%) had ECC, with a mean dmft ± SD of 3.95 ± 3.84. The prevalence of severe ECC was 40.1% (N =269), with a mean dmft of 7.72 ± 3.14. More than 99% of caries were untreated. Statistically significant correlations were found between higher ECC prevalence and increased age and lower socioeconomic background, while greater dental health knowledge of the caregiver and positive oral hygiene behaviors were found to be protective. Our findings confirm the multi-factorial etiology of ECC.

**Conclusions:**

The prevalence of ECC among preschool-aged Uygur children in Kashgar was high, particularly among those from lower socioeconomic backgrounds. Caries prevalence was associated with oral hygiene behaviors of children and the general oral health knowledge of caregivers. These factors could be modified through public health strategies, including effective publicity concerning general dental health and practical health advice.

**Electronic supplementary material:**

The online version of this article (doi:10.1186/1472-6831-14-136) contains supplementary material, which is available to authorized users.

## Background

Dental caries in the primary dentition, known as early childhood caries (ECC) [1], remains a serious public health challenge worldwide, particularly in developing countries [2]. Within-country disparities in caries prevalence among children from different regions and ethnic backgrounds have been widely documented [3–10]. Studies of ethnic minority groups in China have revealed strikingly high caries prevalence among Zhuang, Bonan, Dai, Dongxiang, Korean, Tibetan, and Yugur children [7, 8].

If left untreated, ECC can progress rapidly to devastate the primary dentition [11]. An aggressive subtype, known as Severe Early Childhood Caries (S-ECC), negatively affects children’s physical and mental health [12] and increases the risk of subsequent caries in the permanent dentition [13, 14]. ECC is a preventable disease that cannot be successfully addressed with restorative treatment alone. Risk-based caries prevention and management are important concepts in the study of ECC epidemiology [11].

The classic etiology of ECC involves bacterial, dietary, and host determinants, which are influenced by the interplay of multiple sociological and environmental factors [15, 16]. Studies on risk factors for ECC in different populations have reported various results, including an association between ECC and prolonged breast-feeding [17] and inappropriate feeding practices in very young children [18]. The presence of cariogenic organisms [19], frequency of beverage and food consumption [9, 18, 20], oral hygiene status [21, 22], parental education level, family income [18, 19], caregivers’ oral health knowledge [9, 22, 23], maternal anxiety [24], and child temperament [25] have all been shown to play roles in ECC.

Uygur is one of the major ethnic minority groups in China, with a population of over 10 million people with distinct genetics, customs, culture, and dietary habits [26]. A recent preliminary study found that the prevalence of ECC is higher among Uygur children than among Han Chinese children of the same age in the city of Urumqi [27], which is also higher than the national average [28]. However, epidemiological data about the prevalence of ECC among Uygur children are lacking, and the underlying factors contributing to dental caries among Uygur children remain unclear. Most Uygur individuals live in the less-developed border districts in the rural western area of China, where low socioeconomic status, lack of oral health knowledge, and poor oral health care may contribute to the development of ECC. ECC is a preventable disease, and it is thus necessary to identify the most common and representative predisposing factors.

The objective of this study was to gain a greater epidemiological understanding of the associations between ECC prevalence and relevant socioeconomic, behavioral, and parental conditions among 3–5-year-old Uygur children from the northwest city of Kashgar, China, and to analyze risk factors for ECC development.

## Methods

### Study population

The study was in compliance with the Helsinki Declaration and approved by the ethics committee of the First Affiliated Hospital of Xinjiang Medical University (Xinjiang, China) before dental examination and data collection (Reference number 20130216–103) and was carried out between March and May 2013.

The study was conducted in urban and rural communities in Kashgar, the westernmost city of China, whose population is 92% ethnic Uygur. The drinking water fluoride level in this region is 0.3 ppm. The minimum sample size (n =267) was calculated before study initiation, based on an ECC prevalence of 60% (estimated from the ECC prevalence in the Urumqi population), with a margin of error of 5%, a 95% confidence level, and allowances for a 15% non-response rate, resulting in a required sample size of 313. Initially 730 participants were enrolled in the study. Twenty-nine parents refused to participate in the study because of lack of time. We obtained 697 valid questionnaires; to avoid absence from kindergarten, 27 of these children did not undergo oral examination and were thus excluded. The final analysis included 670 participants, yielding a 92% response rate.

A three-stage stratified sampling method was used to select the study population. Recruitment of the study population was performed with help from the Bureau of Education of Kashgar. There were 17,359 Uygur children aged 3–5 years in 73 kindergartens in Kashgar; 31 kindergartens were in the four urban districts and 42 were in the eight rural townships. In the first stage, Kashgar was stratified into urban and rural areas; three urban districts and six townships were selected randomly from each area. In the second stage, one kindergarten was randomly selected from each urban district or township. In the final stage, one class was randomly selected from each age group (3–5 years) from each kindergarten.

Recruitment strategies included pre-study visits to the selected kindergartens when parents and caregivers arrived to take their children home. We briefly explained the aim of the study to caregivers, and then sent details of the study, including relevant risks, compensation, confidentiality and contact information in a printed form, inviting them to voluntary participate in a questionnaire interview the following day. Written informed consents were obtained from the parents or guardians for the dental health examinations on their children. We provided dental health products, offered oral health recommendations, reported any relevant findings after examinations, and scheduled restorative and preventive treatments for the children.

### Parental interview and data collection

Information on reported ECC risk factors, including (i) demographics (children’s age, gender); (ii) family socioeconomic status (parental education level, family income, residence, family size, parental smoking); and (iii) parent- or caregiver-reported feeding and oral health-related habits, parental dental knowledge, and dental service utilization were obtained via questionnaire (see Additional file 1). The Uygur-language questionnaire was designed by our research team with reference to the guidelines of the American Academy of Pediatric Dentistry and to consensus in the current pediatric dental literature [1, 6, 29]. The questionnaire was pre-tested for clarity. Three trained dental students who speak the Uygur language conducted interviews of parents and caregivers. Data collection was conducted in the kindergarten nurse’s offices. Mothers completed 68.4% of the questionnaires, 25.8% were completed by fathers, and 5.8% by grandparents or aunts.

### Dental health assessment

Two dentists who did not participate in the parental interviews performed the dental examinations. Examiners were trained and calibrated (kappa value, 0.829). The children’s oral health examinations were performed in the kindergarten nurse’s offices with a knee-to-knee posture between 9:30 a.m. and 11:30 a.m. under natural light using disposable plane dental mirrors and explorers. Decayed, missing, and filled primary teeth (dmft) scores were calculated according to World Health Organization criteria [30]. A diagnosis of ECC or S-ECC was made based on the oral health diagnostic criteria defined by the American Academy of Pediatric Dentistry [1]: ECC was diagnosed if at least one tooth was affected by caries. For children between the ages of 3 and 5, the presence of one or more decayed, missing (due to caries), or smooth surfaces in the primary anterior teeth, or a dmft score ≥4 (age 3), ≥5 (age 4), or ≥6 (age 5) was diagnosed as S-ECC.

### Statistical analysis

The presence or absence of ECC was the main outcome variable. General characteristics (age) and caregiver dental health knowledge scores of the caries-free and ECC children were compared with a t-test. Other variables (children’s socioeconomic background, diet, oral hygiene, and dental visit behaviors) related to ECC prevalence were evaluated separately with chi-square tests. Seventeen variables that were statistically significant in univariate analysis, including age, parents’ education level, dental knowledge, family income, family size, feeding habits, dietary habits, and hygiene behavior, were evaluated in logistic models. The forward Wald method was used, with α =0.05 indicating statistical significance. Statistical analyses were performed with SPSS version 17.0 software.

## Results

A total of 670 Uygur children underwent complete dental caries examination and had questionnaires completed by parents or caregivers. The mean age of the children was 4.13 ± 0.80 years; 51.3% were girls and 48.7% were boys. The majority of the children (74.2%) had caries, with a mean dmft ± SD of 3.95 ± 3.84. The prevalence of S-ECC was 40.1% (N =269), with a mean dmft of 7.72 ± 3.14. Most of the caries found were untreated (dt =3.94 ± 3.82; over 99%).

**Figure 1:**
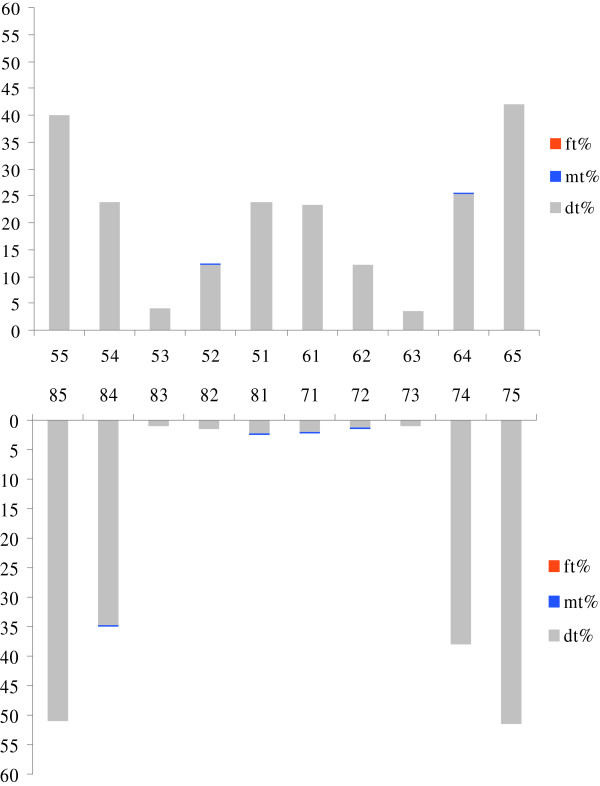
**Distribution of caries experience of children** **(N =** **670)** **.**

Increasing age correlated with increased prevalence and severity of caries. There was no significant correlation between ECC and gender. Prevalence by age and gender is shown in Table 1. The prevalence of dental caries by tooth position is shown in Figure 1. Caries was more often present in the mandibular primary molars than in their maxillary counterparts. In contrast, the mandibular anterior teeth were less affected by dental caries compared with their maxillary counterparts. Canine teeth had the lowest caries prevalence in both the maxillary and mandibular dental arches. The maxillary incisors had a higher caries prevalence than the mandibular incisors (23% versus 2%). Both maxillary and mandibular molars had a high caries prevalence, with the mandibular second molars the most frequently affected teeth. Slightly more than half (51%) of the children had caries in their maxillary posterior teeth.

**Table 1:** **Dental caries prevalence among surveyed children according to age and gender**

	Sample,***n***	ECC,***n (%)***	SECC,***n (%)***	
Age (years)				
3	165	108(65.5)	56(33.8)	χ^2^ for trend = 15.429; p = 0.001
4	239	171(71.5)	97(40.6)	χ^2^ for trend = 3.781; p = 0.052
5	266	218(82.0)	116(43.6)	
Gender				
Male	326	251(50.5)	130(48.3)	χ^2^ = 2.626; p = 0.105
Female	344	246(49.5)	139(51.7)	χ^2^ = 0.051; p = 0.822
**Total** (%)	670(100)	497(74.2)	269(40.1)

Table 2 shows the children’s socioeconomic backgrounds, including mother’s education level, father’s education level, family annual income, and family size. ECC was significantly more prevalent among children from a relatively lower socioeconomic background characterized by lower parental education level (*p* =0.000), lower family income (*p* =0.000), and larger family size (*p* =0.047). While not statistically significant (*p* =0.058), children exposed to cigarette smoke at home were more likely to have ECC.

**Table 2:** **Association between children**’**s socioeconomic background and ECC**

	ECC,***n (%)***	Caries free,***n (%)***	
**Mother**’**s educational level**			
Complete high school	149(61.8)	92(38.2)	χ^2^ = 29.988; p = 0.000
Under high school	348(81.1)	81(18.9)	
**Father**’**s education level**			
Complete high school	130(57.3)	97(42.7)	χ^2^ = 51.257; p = 0.000
Under high school	367(82.8)	76(17.2)	
**Annual household income**			
≥20000 RMB	86(43.7)	111(56.3)	χ^2^ = 135.743; p = 0.000
<20000 RMB	411(86.9)	62(13.1)	
**Family size**			
More than one children	461(75.2)	152(24.8)	χ^2^ = 3.951; p = 0.047
One children	36(63.2)	21(36.8)	
**Smoker in house**			
Yes	167(69.9)	72(30.1)	χ^2^ = 3.594; p = 0.058
No	330(76.6)	101(23.4)	
**Residence**			
Rural	379(74.9)	127(25.1)	χ^2^ = 0.563; p = 0.453
Urban	118(72.0)	46(28.0)	

Table 3 presents children’s feeding history, snacking habits, oral hygiene practices, and dental visit history. A significant association was observed between ECC and the frequency of consumption of sugar-containing soft drinks (*p* =0.000), candy/chocolate (*p* =0.000), fresh fruit (*p* =0.000), sweetened water (*p* =0.000), and milk/yogurt (*p* =0.000). Sleeping with a baby bottle (*p* =0.006), age of initial tooth brushing (*p* =0.000), brushing frequency (*p* =0.000), and parental assistance with tooth brushing (*p* =0.000) were also significantly associated with ECC.

**Table 3:** **Relationship between ECC and children**’**s dietary**, **oral hygiene**, **and dental visit behaviors**

Characteristic	ECC n (%)	ECC-free n (%)	
**Feeding history**			
Breast only	222(74.2)	77(25.8)	χ^2^ = 0.082;
Breast and bottle	250(74.6)	85(25.4)	p = 0.775
Bottle only	25(69.4)	11(30.6)	
Sleep with a bottle			
Always	145(81.9)	32(18.1)	χ^2^ = 7.527;
Never or sometimes	352(71.4)	141(28.6)	p = 0.006
**Dietary habits**			
Sugar-containing soft drink			
Once or less	241(61.3)	152(38.7)	χ^2^ = 82.024;
Twice or more	256(92.4)	21(7.6)	p = 0.000
Cookies⁄cakes			
Once or less	77(53.5)	67(46.5)	χ^2^ = 41.061;
Twice or more	420(79.8)	106(20.2)	p = 0.000
Candy⁄chocolate			
Once or less	120(42.2)	121(57.8)	χ^2^ = 116.865;
Twice or more	377(87.9)	52(12.1)	p = 0.000
Fresh fruit			
Once or less	84(12.5)	115(17.2)	χ^2^ = 151.038;
Twice or more	413(87.7)	58(12.3)	p = 0.000
Sweet added water			
Once or less	199(56.4)	154(43.6)	χ^2^ = 123.489;
Twice or more	298(94.0)	19(6.0)	p = 0.000
Milk/yogurt			
Once or less	75(42.4)	102(57.6)	χ^2^ = 127.049;
Twice or more	422(85.6)	71(14.4)	p = 0.000
**Oral hygiene practices of the children**			
Initial brushing time			
3 years of age or earlier	73(44.5)	91(55.5)	χ^2^ = 99.784;
After 3 years or later	424(83.8)	82(16.2)	p = 0.000
Brushing frequency (times/day)			
twice	5(45.5)	6(54.5)	χ^2^ for trend
once	85(51.8)	79(48.2)	= 61.568;
Some times or never	407(82.2)	88(17.8)	p = 0.000
Parental assistance in tooth brushing			
Yes	169(60.6)	110(39.4)	χ^2^ = 46.205;
No	328(83.9)	63(16.1)	p = 0.000
**Dental service utilization**			
Visited a dentist			
Yes	21(53.8)	18(46.2)	χ^2^ = 8.938;
No	476(75.4)	155(24.6)	p = 0.003
Prenatal dental health advice history			
Yes	71(74.0)	25(26.0)	χ^2^ = 0.003;
No	426(74.2)	148(25.8)	p = 0.957
Caregiver’s general dental knowledge score	4.64 ± 1.36	4.65 ± 1.27	p = 0.345

The prevalence of ECC was higher among those who had never had professional dental care (631 children; 95.8% of those with ECC and 89.6% of caries-free children; *p* =0.003). When asked about barriers to dental healthcare, 333 caregivers (41.7% of those in the ECC group and 18.2% of those in the caries-free group) indicated that their children had no dental health problems. Fifty-nine caregivers (8.5% of those in the ECC group and 2.2% of those in the caries-free group) indicated that the problem was not serious enough to visit a dentist. Seventy-four caregivers (9.4% of those in the ECC group and 4.0% of those in the caries-free group) indicated that treatment of deciduous teeth is not necessary. Eight caregivers (1.3% of those in the ECC group and 0.2% of those in the caries-free group) indicated that it is very costly to treat the deciduous teeth. Thirty-one caregivers (4.3% of those in the ECC group and 1.3% of those in the caries-free group) indicated that their child did not want to visit a dentist. Fourteen caregivers (2.2% of those in the ECC group and 0.4% of those in the caries-free group) indicated that they had no time to take their child to a dentist. Other reasons for not pursuing dental care included difficulty finding nearby dental clinics.

Caregivers’ general dental health knowledge was assessed based on answers to 10 questions about early childhood dental health. These questions concerned the importance of primary teeth and good dental health, well-known general causes of dental caries, and caries prevention knowledge [1, 6, 29]. Agreement with a correct answer earned one point; no points were awarded for wrong or uncertain answers. There was no significant difference between the mean knowledge scores of the two groups. The overall level of dental knowledge was not high in this study population, with a mean knowledge score of 4.64 ± 1.34 (range, 0–10), although the information in the survey is thought to be common knowledge, given public exposure to mass media. Only 14.3% of surveyed mothers had received prenatal dental health advice. Most caregivers admitted that their dental health knowledge was obtained from product advertising and newspapers, rather than from professionals.

Table 4 shows the results of logistic regression model analysis, which offers an accurate representation of the interplay of ECC-related determinants. The prevalence of ECC significantly increased with age (OR =1.438; 95% CI =1.031–2.006) and was significantly higher among those from lower-income families (OR =2.858; 95% CI =1.611–5.049). High-frequency consumption of fresh fruit (OR =3.337; 95% CI =1.920–5.799), sweetened water (OR =3.356; 95% CI =1.658–6.794), or milk/yogurt (OR =3.039; 95% CI =1.704–5.420) was also associated with higher ECC prevalence. Greater caregiver dental health knowledge (OR =0.740; 95% CI =0.606–0.902), younger age at initial tooth brushing (OR =0.400; 95% CI =0.227–0.707), and higher brushing frequency (OR =0.449; 95% CI =0.606–0.902) were protective against ECC.

**Table 4:** **Logistic regression analysis of ECC risk determinants**

	OR	SE	95%CI	P
Age	1.438	0.170	1.031-2.006	p = 0.032
Fresh fruit	3.337	0.282	1.920-5.799	p = 0.000
Sweet added water	3.356	0.360	1.658-6.794	p = 0.002
Milk/yogurt	3.039	0.295	1.704-5.420	p = 0.000
Annual household income	2.858	0.291	1.611-5.049	p = 0.000
Initial brushing time	0.400	0.290	0.227-0.707	p = 0.002
Brushing frequency	0.449	0.285	0.257-0.785	p = 0.005
Caregiver’s dental health knowledge	0.740	0.107	0.606-0.902	p = 0.003

## Discussion

Children are a disadvantaged population predisposed to dental caries, which negatively affects quality of life of young children around the world [12, 31]. This study provides much-needed information on the dental caries condition among Uygur preschool children residing in a westernmost city in China. Most Uygur people in China live in southern Xinjiang Province. Kashgar is an ancient city in south Xinjiang whose population is 92% ethnic Uygur [26]. Because preschool education is funded by China’s central government, 92% of preschool-aged children are enrolled in daycare in Kashgar. The study population enrolled in the present survey was selected through multi-stage sampling, which provided a representative and suitable sample.

We found that the prevalence of ECC among 3–5-year-old Uygur children in Kashgar was similar to that reported in our previous survey of children in Urumqi [27]. ECC was found in 74.2% of the enrolled children and S-ECC was diagnosed in 40.1%. The prevalence and severity of dental caries in these children increased with age, probably because of longer exposure to environmental cariogenic challenges, as reported in other studies [3, 32]. The caries prevalence in this study was high compared with that in other populations around the world [4, 7, 22, 33, 34]. Moreover, over 99% of affected children remain untreated. Only 5.8% of the surveyed children had received professional dental care, most often for treatment of pain and abscesses. These results are similar to those of other studies, which found that professional care was sought only for symptomatic ECC [32, 35]. When asked about barriers to dental healthcare, over half (59.8%) of parents responded that there was no problem with their children’s teeth, even when their children suffered from severe dental caries. These findings indicate that parents and caregivers are unaware of the dental health status of their children and of the need for treatment until children have severe symptoms. The high caries prevalence, severity, and lack of treatment in this study highlight the need for dental health promotion programs to prevent ECC in this population.

This study sought to reveal the most common and representative barriers to good dental health among preschool-aged Uygur children in Kashgar. We identified many factors, some of which are related to family socioeconomic situation and some to caregivers’ knowledge about proper health behavior choices. Most parents were not concerned about their children’s deciduous teeth, were unaware of their children’s dental health condition, and did not address the dental disease present in their children. Fluoride toothpastes are available for purchase in China, but few parents (15.2%) knew about its role in preventing dental caries, so their effects have not been evaluated. The language barrier, lack of dental health material in the Uygur language, and ethnic oral health beliefs may contribute to caregivers’ low level of dental knowledge. Traditional Uygur medicine promotes balance in the human body; rock sugar or granulated sugar is added to water based on its characteristics, which may be another reason for the severe caries status in this population.

Socioeconomic status relating to family income and parents’ educational attainment is a widely documented risk factor for ECC [3, 4, 15]. We found that children from relatively low-income families had a higher risk of ECC. However, logistic regression analysis revealed no obvious correlation between parents’ educational attainment and ECC, unlike the findings of studies in other populations [18, 19, 36], possibly because parental educational level was relatively low and homogeneous in this study population. Parents and caregivers have a responsibility for children’s health status, including oral health. Caregivers play a central role in determining children’s dental health behaviors, include tooth brushing, dental care, and dietary habits, which are acknowledged to be protective factors for primary teeth [33]. Supervised tooth brushing with fluoride toothpaste has been associated with a 20–30% reduction in caries prevalence in other countries [34]. Lack of familiarity with children’s dental health condition, low general oral health knowledge, and negative dental disease preventive behaviors were revealed in this population, which might be attributable to parental educational qualifications and to socioeconomic and cultural status, as previously observed [18, 23, 29, 37]. Parents also underestimate the importance of deciduous teeth, because they are replaced by permanent teeth. Our investigation found that only 14.3% of surveyed mothers had received prenatal dental health advice and most caregivers reported that they obtained dental health information from television advertisement rather than from professionals.

There is no dental treatment insurance program for children in China; individuals pay for all costs without government subsidy. However, preventive dental health intervention projects for children, including topical fluoride application and sealants for pits and fissures, have been undertaken with special government funding. The finding that unprofessional sources provide most oral health information for this study population and the low utilization of efficient technology confirm that there has been no significant improvement in access to appropriate oral health advice and preventive dental care. It is important to provide clear and appropriate dental health advice to improve caregiver awareness of their children’s dental health status and of their pivotal role in ECC prevention.

Unlike previous studies in which the highest caries prevalence was found in the maxillary incisors [6, 7], we found that mandibular primary second molars were the most frequently affected teeth, followed by maxillary second molars. Deep anatomic grooves, which are prone to food retention, combined with poor oral hygiene habits and a lack of preventive measures, makes the teeth more susceptible, especially to pit and fissure caries [38].

This was a cross-sectional study, and thus does not allow determination of cause–effect relationships. While this study gives us a glimpse into the dental condition among Uygur children in west China and the most important factors associated with ECC in this population, the study has several limitations. First of all, selection bias is possible, because only 670 subjects were selected from over 10,000 eligible children. We tried to minimize this bias by selecting kindergartens from almost all districts of Kashgar. Furthermore, the data provided by caregivers were retrospective; caregivers may have responded with the intention of pleasing the interviewer or may have been guided by the interviewers during the questionnaire, leading to potential response bias. Nonetheless, our findings reveal ECC and S-ECC prevalence and factors related to the condition. Awareness of ECC prevalence and interventions is most important in designing preventive public health strategies. Multiple interventions with community and professional management programs are needed to prevent ECC [39]. Development of educational programs targeting low socioeconomic groups and providing regular medical care, especially for women of low socioeconomic status before and during pregnancy, is necessary [1, 40]. Additional measures include early screening for signs of ECC; risk assessment in high-risk groups; anticipatory guidance for parental intervention, including hygiene instruction and limiting children’s exposure to sweet beverages; and professional preventive intervention programs that include topical fluoride application, pit-fissure sealants, and special treatment as needed [41, 42]. The Department of Public Health should propose and implement projects to bridge the gap in oral health between privileged and disadvantaged children. Improvements in education, employment, and living standards would probably help improve the situation.

## Conclusions

A high caries prevalence and severity and a lack of caries treatment were revealed among Uygur preschool children in this study. Frequent exposure to readily accessible fruit, sweetened water, and milk, along with poor oral hygiene habits and a lack of preventive measures, contribute to the negative dental health condition in this population. Lack of awareness of their children’s dental health status, unprofessional sources of oral health information, and low utilization of efficient technology contributed to the poor general oral health knowledge of caregivers. These factors could be modified through public health strategies, such as effective publicity concerning general dental health and practical health advice.

## Electronic supplementary material

Additional file 1: **Oral health survey of children in Kashgar.** (DOC 54 KB)
