# Supplementary material for: Risk determinants associated with early childhood caries in Uygur children: a preschool-based cross-sectional study
Source: BMC Oral Health. 2014 Nov 18;14:136. doi: 10.1186/1472-6831-14-136 (PMC4242481; doi:10.1186/1472-6831-14-136)
Supplement: Supplementary file 1 — Additional file 1: Oral health survey of children in Kashgar. (DOC 54 KB) [file 12903_2014_465_MOESM1_ESM.doc]

Date: ____/____/______ (DD / MM / YYYY) Case Number:

**Oral health survey of children in Kashgar**

Dear parents:

This questionnaire is important for considerations to mitigate child’s risk of early childhood caries. We ask your serious completion. Thank you for your cooperation.

**Child name:**  **Gender:** **□ Male □ Female**

**Birth date: : ____/____/______ (DD / MM / YYYY) Contact number：________________**

1. You are child’s : □ Mother □ Father □ Grandparents □ Other relatives
2. How many children do you have?

□ One □ More than one

1. Do child’s parents smoke?

□ Yes □ No

1. Parent education level:

Father □ Complete high school □ Under high school

Mother □ Complete high school □ Under high school

1. Annual household income:
2. How to feed your child during her/his first year of life?

□ Breast only □ Breast and bottle □ Bottle only

1. Is your child sleep with a baby bottle?

□ Always □ Sometimes or never

1. Other than meals, how often does your child snack?

Soft drinks:

□ None □ 1 times □ More than 2 times

Cookies, cakes:

□ None □ 1 times □ More than 2 times

Candy, chocolate:

□ None □ 1 times □ More than 2 times

Fresh fruit

□ None □ 1 times □ More than 2 times

Sweet added water

□ None □ 1 times □ More than 2 times

Milk/yogurt

□ None □ 1 times □ More than 2 times

9. When did your child start brushing teeth?

□ Before 12 months old □ 13 to 24 months old □ 24 to 36 months old □ After 24 months old

□ Not yet

10. How many times does your child brush everyday?

□ Never □ Seldom □ Once □ Two times or more

11. Does anyone assist your child in brushing his/her teeth?

□ Yes □ No

12. Have your child ever seen a dentist?

□ Yes □ Never

**If “Yes”**, what is the reason for visit a dentist?

□ Traumatic dental injuries

□ Pain

□ Abscess

□ Dental check up

□ Preventive treatment

□ Other

**If “No”**, what is the reason for never visit a dentist?

□ I can’t find dental care nearby

□ There is no dental health problem with my child

□ The problem is not serious enough to visit a dentist

□ It’s not necessary to treat the baby teeth

□ The treatment is very costly

□ My child is afraid of dentist

□ I have no time to take child for visit

13. The main source of your oral health knowledge：

□ Community health care center

□ Dentist

□ Hospital

□ Friends and relatives

□ Television advertisement

□ Internet

□ News paper and health magazine

14. Did child’s mother have prenatal dental health advice?

□ Yes □ No

15. Baby teeth are important even though they fall out.

□ Agree □ Not agree

Problems with baby teeth will affect adult teeth.

□ Agree □ Not agree

Tooth decay could affect child’s health.

□ Agree □ Not agree

Regular dental visit is necessary for children.

□ Agree □ Not agree

Dentist can help prevent tooth decay.

□ Agree □ Not agree

Tooth brushing should be performed twice daily.

□ Agree □ Not agree

It’s okay to put my child to bed with a bottle.

□ Agree □ Not agree

Frequent consumption of sweet causes dental decay.

□ Yes □ No □ I don’t know

Brushing teeth will help prevent tooth decay.

□ Yes □ No □ I don’t know

Fluoridated toothpaste will help prevent tooth decay.

□ Yes □ No □ I don’t know

- The End-
